# A non-linear mathematical model using optical sensor to predict heart decellularization efficacy

**DOI:** 10.1038/s41598-019-48659-3

**Published:** 2019-08-21

**Authors:** Rayssa Helena Arruda Pereira, Adilson Ribeiro Prado, Luiz Felipe Castello Del Caro, Tadeu Ériton Caliman Zanardo, Airlane Pereira Alencar, Breno Valentim Nogueira

**Affiliations:** 1 Carlos Alberto Redins Cell Ultrastructure Laboratory (LUCCAR) and Tissue Engineering Core, Department of Morphology - Health Sciences Center, Federal University of Espírito Santo (UFES), Vitória, ES Brazil; 2 Biotechnology Graduate Program - Rede Nordeste de Biotecnologia (RENORBIO), Vitória, ES Brazil; 3 Department of Control Engineering and Automation, Federal Institute of Espírito Santo, Serra, ES Brazil; 4 Department of Statistic, Institute of Mathematics and Statics, São Paulo University, São Paulo, SP Brazil

**Keywords:** Tissue engineering, Cardiac device therapy, Optical sensors

## Abstract

One of the main problems of the decellularization technique is the subjectivity of the final evaluation of its efficacy in individual organs. This problem can result in restricted cell repopulation reproducibility and worse responses to transplant tissues. Our proposal is to analyze the optical profiles produced by hearts during perfusion decellularization, as an additional method for evaluating the decellularization process of each individual organ. An apparatus comprised of a structured LED source and photo detector on an adjustable base was developed to capture the relationship between transmitted light during the perfusion of murine hearts, and residual DNA content. Voltage-time graphic records were used to identify a nonlinear mathematical model to discriminate between decellularizations with remaining DNA above (Incomplete Decellularization) and below (Complete Decellularization) the standardized limits. The results indicate that temporal optical evaluation of the process enables inefficient cell removal to be predicted in the initial stages, regardless of the apparent transparency of the organ. Our open system also creates new possibilities to add distinct photo detectors, such as for specific wavelengths, image acquisition, and physical-chemical evaluation of the scaffold, in order to collect different kinds of information, from dozens of studies. These data, when compiled and submitted to machine learning techniques, have the potential to initiate an exponential advance in tissue bioengineering research.

## Introduction

Studies have demonstrated that 40–50% of transplanted organs are lost within ten to twelve years, and a high proportion of the remainder suffer from side effects related to immune suppression^1,2^. Thus, the organ and tissue decellularization process was developed not only to provide a greater supply of organs for transplantation, but also in an attempt to prevent rejection reactions^3^. Despite the complexity and multiplicity of effector mechanisms, rejection reactions can typically be triggered by the presence of antigens such as natural donor isohemagglutinins, leukocytes or allelic alloantigens (minor histocompatibility or non-HLA antigens)^4–6^. Moreover, the release of mitochondrial DNA into the circulation can also activate immune responses^7^. Consequently, efficient removal of the donor’s original cells, as well as any cellular material, including DNA, is required to minimize or even avoid triggering such reactions^8^.

This concept guided the development of the decellularization technique by conferring low immunogenicity and also producing scaffolds with unique structure and properties^9–12^. The decellularization process must be capable of preserving the original extracellular matrix (ECM), which is essential for maintaining the three-dimensional geometry of the organ, and on which the recipient multipotent stem cells will be cultured, through cell repopulation^13^. Complex organs, such as the heart^14^, liver^15^ and kidneys^16^, is still in the experimental phase and has presented great potential for progression to clinical trials in humans^3,17^. The process of obtaining decellularized organs is based on cell removal through the use of surfactants, either alone or in association with biological agents, such as enzymes; chemical agents, such as acids and bases; or physical agents, such as sequential freezing and thawing^10,11^. Since the cells consist mainly of lipids in their membranes and endomembranes^18^, the surfactant action disrupts the organization of the cell membranes, breaking down the cells, nuclei and organelles. The pressure exerted by the continuous organ perfusion flow carries the disorganized cellular material to the outside of the organ through its own vascular network. This process is termed perfusion decellularization, and produces acellular scaffolds^19^.

There are some limiting factors that need to be overcome, such as the predictability and consequent reproducibility of organ decellularization, and the lack of objective, specific and individual criteria for determining successful decellularizations^20,21^. A successful decellularization is one in which the amount of remaining DNA is 50 ng.mg^−1^ of dry tissue^8^, or in which DNA removal is about 96% when compared to the control organs^11^, and in which the three-dimensional structure, especially the vascular structure, is maintained after the process. Nowadays, it is widely accepted that a successful decellularization can be confirmed by histological analysis with DAPI or Hematoxylin and Eosin (H&E), an observed lack of visible nuclear material, and through spectrophotometric quantification of DNA, collagen and glycosaminoglycans^8,11,22–25^. But these criteria are associated with destructive methods at the end point of the decellularization process, which creates an obstacle to assess the organs individually before recellularization without causing them damage. As a result, only visual and temporal standardization of the final evaluation of the individual process, i.e. verification of a translucent color, with clear visualization of the vascular network, are possible, without destroying the organ^11^. But these criteria may be affected by individual characteristics of the organ, including intrinsic morphological aspects such as weight and age; or procedural errors, that may go undetected^11,26^.

In view of the above, and considering the lack of a gold standard based on non-destructive alternatives for organs during or post-decellularization, our group developed a system that captures the photons transmitted through the organ and emitted by a light emitting diode (LED), converting them into voltage values in real time and collecting the values for the individual temporal study of each decellularization. Despite standardization of the decellularization protocol, simultaneous optical monitoring has demonstrated that there is an optical profile for each individual heart decellularization, which can be correlated with residual DNA concentrations. Thus, we have developed a standard of quality that can predict the inefficiency of cell removal in the early stages of the decellularization process. This will enable inefficient decellularizations to be identified, even where there is apparent organ transparency, providing an exponential advance in tissue bioengineering research.

## Results

### DNA quantification, collagen and histological analyses and heart mass

**Figure 1:**
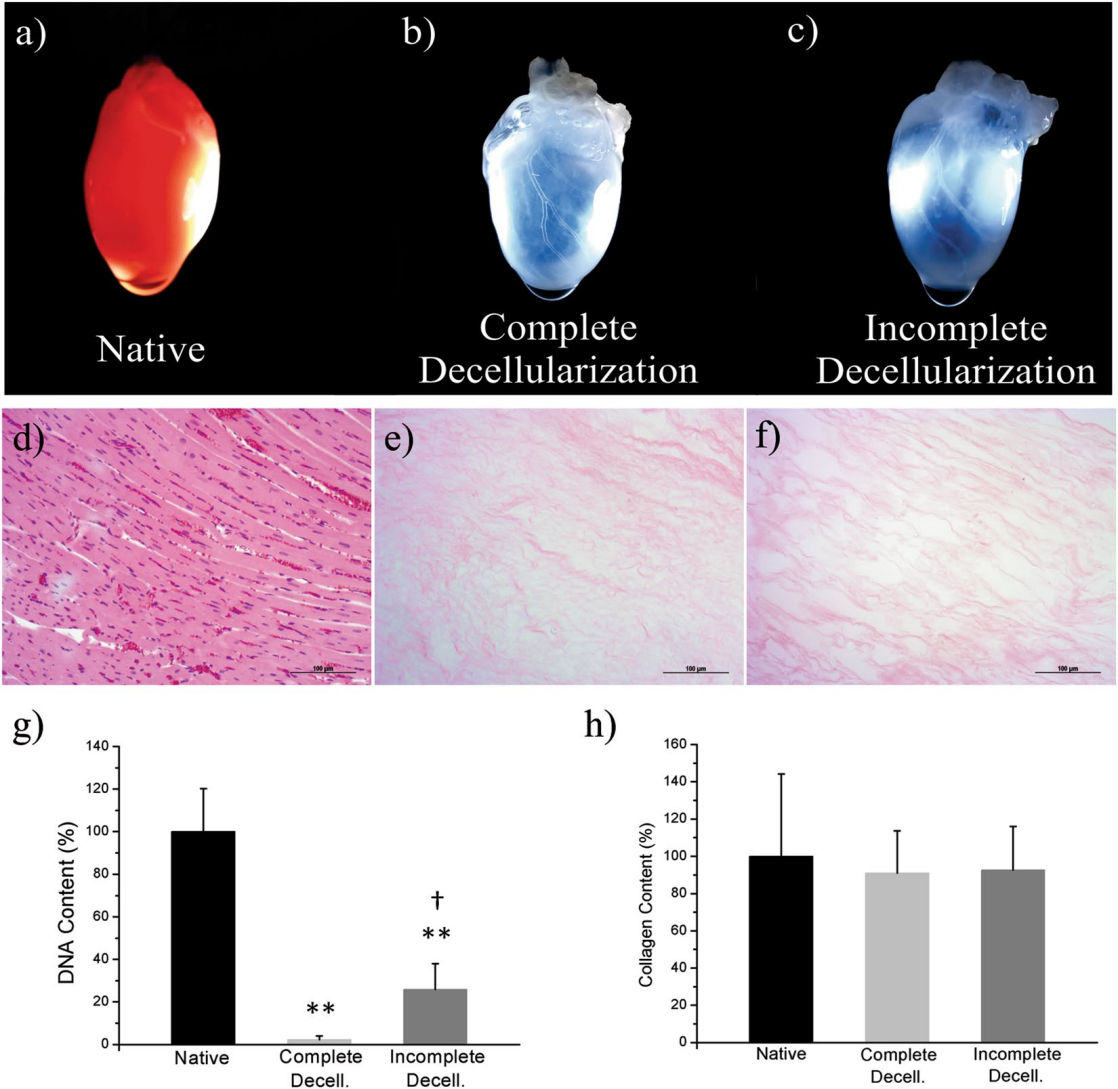
Representative pictures of hearts, their respective total DNA and collagen content; and, H&E staining of histological sections. (**a**) Picture of a native rat heart. (**b**) A rat heart from the Complete Decellularization Group. (**c**) A rat heart from the Incomplete Decellularization Group (relative to curve 7, depicted in Supplementary Fig. 3b). (**d**–**f**) Representative H&E staining histological sections of Native, Complete Decellularization and Incomplete Decellularization groups. (**g**) Bar graphs of DNA percentage of lyophilized heart from Native (n = 6), Complete Decellularization (n = 6) and Incomplete Decellularization (n = 8). **p = 0.0002 *vs*. Native group; **†***p* = 0.0036 *vs* Decellularized group. Values are mean ± SD. (**h**) Collagen content of lyophilized heart from the Native (n = 4), Complete Decellularization (n = 4) and Incomplete Decellularization (n = 4) groups, *p* > 0.05.

All the studied decellularizations were submitted to the same protocol. White transparency was confirmed, with clear visualization of the vascular ducts, as shown in Fig. 1(a–c). In addition, the organs were almost transparent when the asymptote curve was reached. Thereafter, subtle changes occurred gradually, until the last hour, when no macroscopic changes were visualized.

Each individual decellularization was subsequently categorized as Complete Decellularization or Incomplete Decellularization, according to the results of the DNA concentration (Fig. 1g): for the Complete Decellularization group, the DNA concentrations were within the values accepted by the scientific community, and for the Incomplete Decellularization group, the residual DNA values were outside those values, and therefore unacceptable.

The Complete Decellularization group consisted of cases where the proportion of DNA remained below 4.6% when compared to the control organs, corresponding to an average of 27 (±19 SD) ng of DNA per mg of extracellular matrix dry weight and a percentage average of 2.35% (±1.63% SD). Meanwhile, the Incomplete Decellularization group presented an average DNA concentration of 301 (±141 SD) ng.mg^−1^, represented by a percentage average of 25.87% (±12.07% SD). DNA concentration was therefore chosen as the response variable, due to its high correlation with the optical profile. This finding confirms the sensitivity of DNA quantification for evaluating cell removal efficacy.

H&E histological evaluation and collagen quantification were performed to confirm the decellularization. Histological evaluation revealed an exposed extracellular matrix showing no nuclear staining after whole organ decellularization for both Complete and Incomplete Decellularization samples (Fig. 1e,f). The difference in collagen content was not significant between native hearts and those submitted to decellularization; the difference was also not significant between the Complete and Incomplete Decellularization groups (Fig. 1h).

When assessing the distribution of animal masses and fresh organ masses between the Complete Decellularization and Incomplete Decellularization groups, no significant difference was found between them (Supplementary Fig. 1a,b). The dry weight of the native hearts was reduced by around 87% after all decellularizations, from an average of 232 ± 9.1 mg to 29 ± 6.8 mg (p < 0.001). But when comparing the lyophilized mass in the Complete Decellularization and Incomplete Decellularization groups after the decellularization process, the difference between averages was not significant, with an average of 23 ± 5.5 mg for Complete Decellularization and 33 ± 5.9 mg for Incomplete Decellularization (Supplementary Fig. 1c).

### Decellularization monitoring optical system

**Figure 2:**
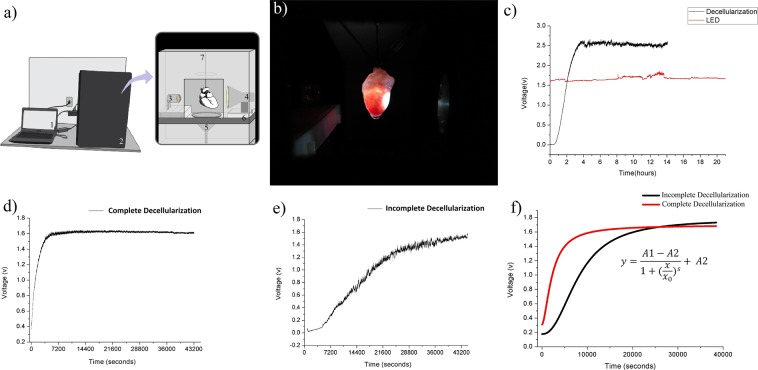
Schematic diagram of the optical sensor experimental setup, representative optical profile of each group and estimated typical curves for each group. (**a**) Schematic diagram of the monitoring system experimental setup: computer and open-source electronic prototyping platform (1) connected to the dark chamber (2), that accommodates the monitoring system. The monitoring system components are the photo detector (3), the light source (4), the discard system (5), the three axes adjustable base (6) and the infusion hose (7) connected to the perfusion pump that stay beside to the chamber. The organ stays between the photo detector and the light source. (**b**) Picture of the optical monitoring system during a decellularization process. (**c**) Monitoring of the isolated LED source for 20 hours, compared with a decellularization process monitoring. (**d**) Monitoring of a Decellularized group sample (curve 2 of Supplementary Fig. 2a). (**e**) Monitoring of an Incomplete Decellularization group sample (curve 2 of Supplementary Fig. 2b). (**f**) Typical “Voltage x Time” curves of each group, Complete Decellularization (n = 6) and Incomplete Decellularization (n = 8), fitted by nlme version 3.1 from Pinheiro and Bates^28^ in software R with the four-parametric non linear model described by Gadagkar and Call^27^.

The voltage measurement profile along time was based on photon detection by a wide-detection photodiode, aligned with a white LED source. The organ was placed in the center, by a three-axes alignment system that lies inside a dark chamber connected to a computer (Fig. 2a,b). The measurement of isolated LED monitoring, compared to decellularization monitoring, is shown in Fig. 2(c). The real-time recordings observed for each group are shown in Fig. 2(d,e). The decellularizations recorded by the optical system were satisfactorily modeled by a four parametric non linear model described by Gadagkar and Call, 2000^27^; the typical estimated behaviors for each group and the equation are shown in Fig. 2(f).

### Mathematical model of the optical profile

The mixed non linear model was fitted and the equation parameters estimated, using the R package nlme (Linear and Nonlinear Mixed Effects Models, version 3.1) described by Pinheiro and Bates^28^. The initial voltage (A1), asymptote (A2), inflection point (x_0_) and curve slope estimates (+/**−** standard errors) for the Complete Decellularization group were A1 = 0.3100, A2 = 1.6946 (±0.0026), x_0_ = 2067.8359 (±32.5140) and *s* = 1.6530 (±0.0288), respectively, and for the Incomplete Decellularization group, A1 = 0.1779, A2 = 1.7760 (±0.0048), x_0_ = 7352.9140 (±50.4550) and *s* = 2.2710 (±0.0335), respectively. There was a significant difference between groups for the parameters A1, A2 and *s* (p < 0.001). However, despite the difference between estimates, the inflexion point (x_0_) showed wide variances within groups, thus these differences were non-significant (p = 0.142).

All the observed curves are shown in Supplementary Fig. 2(a,b) for the Complete Decellularization and Incomplete Decellularization groups. The Incomplete Decellularization group (Supplementary Fig. 2b) had larger variations than the Complete Decellularization group (Supplementary Fig. 2a). This is because there are more levels of inefficient processes than efficient ones.

### Optical profile spectral analysis

**Figure 3:**
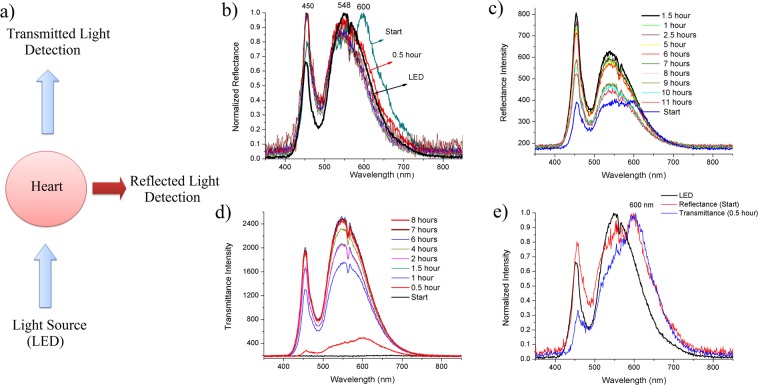
Spectral analysis of the decellularization process using the fiber optic spectrophotometer. (**a**) Schematic diagram of the setup of spectral measurements. (**b**) Normalized intensities (0–1) of the reflectance spectra versus wavelength obtained during the perfusion with SDS and the LED spectrum. (**c**) Absolute intensities of reflectance spectra demonstrating maximum values at between 1 and 2 hours. (**d**) Absolute intensities of transmittance spectra showing an overall increase over time. (**e**) Normalized (0–1) spectra of reflectance at the initial moment of SDS perfusion, and transmittance at 30 minutes of SDS perfusion, compared with the light source profile (LED), evidencing a band shift at 600 nm.

For the spectral analysis, two optic fibers connected to an USB spectrophotometer were placed at 180° and 90° from the light source position to record the transmittance and reflectance effects. A schematic diagram of the experimental setup is shown in Fig. 3(a). The relative reflectance intensity at 450 nm increased during the decellularization process and a displaced band at 600 nm disappeared in the first hour (Fig. 3b). The maximum value of the intensity of absolute reflectance occurred at between 1–2 hours of SDS perfusion (Fig. 3c). The transmittance intensity increased over time (Fig. 3d). When compared, both the transmittance and reflectance spectra showed a displaced band at 600 nm (Fig. 3e).

## Discussion

The present study demonstrates, for the first time, the relationship between extent of cell removal and optical profile of transmitted light through the heart during perfusion decellularization. This enabled the proposal and estimation of a mathematical model capable of discriminating between decellularizations with DNA discharge above and below the recommended limits.

Our absolute amount of DNA within the Complete Decellularization group findings was similar to those observed in the literature for the use of SDS detergent, either isolated or accompanied by proteolytic enzymes, represented by a percentage average of remaining DNA of 2% when compared to cadaveric murine hearts^11,13^. The categorization of the group simultaneously followed the main current standards for maximum remaining DNA, i.e., approximately 50 ng.mg^−1^ of dry tissue^8^ or residual DNA content of approximately 4%, when compared to the control organs^11^.

The optical profile produced by decellularization of rat hearts, using the white light source, was satisfactorily described by the regression model first proposed by Hill, 1910^29^, with later modifications by Gadagkar and Call, 2015^27^. This S-shaped model is justified by the high extinction of light at practically all wavelengths, which occurs in the first instance by tissue-light interaction, and decays as the main endogenous chromophores, such as hemoglobin and myoglobin (absorption range of 400–600 nm) and fat^30,31^ are extracted, before finally reaching a plateau. This is shown in Fig. 3d, which demonstrates the low perceived intensity at the beginning of the transmittance spectrum due to photon scattering, absorption, reflectance and total internal reflection^30^. These photons, detected at the 30-minute transmittance spectrum, based on the organ opacity at this time point, relate to the initial transmitted radiation, as well as the emission of autofluorescent structures and molecules, such as collagen cross-links (400–500 nm)^22^, NADH (~450 nm), FAD (515 nm)^31^ and, in particular, 600 nm-emission molecules such as porphyrins (emission range between 580–640 nm)^32,33^.

The emission hypothesis is also confirmed by the reflectance spectra, which demonstrated a displaced band at 600 nm in the first hour (Fig. 3a). The remaining wavelength signals were probably influenced by scatterings, reflections, emissions and absorption interferences. Thus, our data indicate that the porphyrin discharge rate is one of the main factors responsible for the initial behavior of the optical profile, and indicates precise moment at which the extent of cell removal by decellularization can be predicted. The fluorescence decrease rate at 600 nm is, therefore, an important variable to be modeled and aggregated to the algorithm, which is in constant development. Moreover, the monochromaticity also will allow greater selectivity of fluorophore excitation, absorption and scatterings, demonstrating that improvements to the system can yield more useful data for real time evaluation of the decellularization process.

In relation to the evaluation of fitted curves, represented in Fig. 2f, the Complete Decellularization group showed initial and final voltage intervals with a lower range amplitude than the Incomplete Decellularization group, this amplitude being partially determined by the slope coefficient (*s*). The *s* parameter, usually called the Hill coefficient^29^, presented significant difference between groups. Higher positive *s* values induced a faster increase towards the asymptote, eliminating the initial part of the S-curve (Supplementary Fig. 3). The estimates of the halfway time between minimum and maximum voltage asymptote (x_0_) were very different for both groups: two hours for Incomplete Decellularizations, as opposed to thirty-five minutes for the Complete Decellularization group. This may suggest that with inefficient processes, more time is needed to reach the inflexion point, but this analysis still requires prior treatment to reduce signal noise in order to reduce the variability of this parameter estimate for both groups.

It is important to point out that the Incomplete Decellularization group showed greater variability, as shown by the standard errors of the estimators and by observation of the individual curves (Supplementary Fig. 2). There is one curve in the Incomplete Decellularization group, for example (Supplementary Fig. 2b; Curve Number 6), that presented smaller growth rate than all the others, representing a decellularization process in which the cell removal occurred slowly. However, in general, the curves presented a lower growth rate in the first hour, followed by a sharp rise, which was not observed in the Complete Decellularization group. On the other hand, the Complete Decellularizaton group demonstrated homogeneity, despite the presence of electrical noise (Supplementary Fig. 2a) that may be due to terminal voltage fluctuation of the light emitting diode, fluctuations in flow current, fluctuations in optical noise from the LED light output^34^, and the influence of the medium on the optical path. This is currently being investigated by our team.

The present study therefore demonstrates that the optical profile acquired through a simple optical monitoring system is able of predicting the inefficiency of cell removal in the first hours of SDS perfusion, allowing the researcher to stop the process and find the errors, avoiding wasted time and reagents. This early pattern indicates that an initial inadequate perfusion configuration should be investigated as possible causes of failure, rather than later events that may occur during the process. In our experience, possible factors that trigger inadequate perfusions include intrinsic individual vascular characteristics related to the pattern of cellular debris accumulation in the lumen^26^, inherent organ changes due to disease or age^11^ and procedural errors such as very deep positioning of the cannula in the aorta, organ perforations not observed and possibly committed at the time of adjacent tissue removal, the presence of impurities in the infusion hoses, connections, needles or cannulas, acting as nucleation points for the formation of protein clusters, and the presence of blood micro-clots that were not perceptible in the visual identification.

Our study also evidenced that each heart has its own unique decellularization optical profile. It also demonstrated that visualization of extent of white transparency, or following a precise procedure time, are not necessarily guarantees of a successful decellularization. The same was observed for the apparent stabilization of the graphs, which was attributable not to the end of the decellularization process, but to a phase of slow removal of cellular residuals in small concentrations. Therefore, analysis of the graph profile, supported by the non-linear model description, is essential, since this can determine inefficient cell removal without destroying the sample, as occurs with DNA quantification procedures.

In this work, the efficacy of decellularization was considered in terms of the extent of cell removal, and was demonstrated by DNA quantification, due to its quantitative characteristic, high sensitivity, and the representativeness of the portion of tissue analyzed, as well as being a strong quality control criteria for tissue disapproval after decellularization^8,24,35^.

H&E microscopy visualization and collagen quantification were not capable of differentiating between Complete Decellularization and Incomplete Decellularization groups (Fig. 1d–f, h). This was to be expected, considering other findings of non-significant changes in collagen, and even in sulfated glycosaminoglycan content between decellularized and native organs, using standardized protocols^23,36^.

DNA quantification enables a considerably greater tissue mass to be evaluated than is possible with conventional histological analysis. This greater tissue mass can be estimated by assuming a possibility of 3 thousand 5-micron sections in 15 millimeters of rat heart, at its greatest diameter, in which 50 sections of each sample can represent only 1.5% of whole organ. In contrast, DNA analysis, using the spectrophotometric technique, showed representativeness of up to 40% of the total mass of lyophilized and decellularized organs, in an analysis of samples of up to 14 mg of tissue.

The quality control of decellularized extracellular matrices has recently received great attention, due to the lack of standard methods and alternatives for non-destructive characterization. Quinn and co-workers^22^ demonstrated that second harmonic generation (SHG) and non-destructive multi-photon imaging could predict elastin and collagen behavior and mechanical properties after myocardium infarction in decellularized extracellular matrices. These findings open up new possibilities for the use of diversified types of optical data in order to standardize extracellular matrices after decellularization, and these data may be used alongside our optical system. Raman Scattering Spectroscopy– RS^37^ is another nondestructive alternative for tissue engineering, and can identify changes in signal intensity that indicate changes in the composition and proportions of matrix components in cardiac tissues. At its current stage of development, research in RS is still focused on tissue evaluation, but it has demonstrated potential for application to whole organ decellularization in the future.

Other examples are X-ray Phase Contrast Computed Tomography - PC-CT^38^ and Microcomputed Tomography - µ-CT^39^ both of which, when focusing on tissue constructs with highly complex geometries and high quality visualization, can be applied to tissues or organs fragments; and, when the goal is to assess density, Computed Tomography – CT showed potential for application to whole organs. Geerts and co-authors^40^ demonstrated that rat livers could be monitored using non-contrast CT scan, in which Hounsfield units (HU) could be applied to identify the extent of cell removal, with good correlation and potential to scale-up to larger organs. However, those authors have also shown that there are some challenges to be overcome before HU can be implemented in isolation, such as sensitivity, which was of the order of up to 0.2 μg.mg^−1^of DNA, using wet weight, at the lowest value of HU, being considered less sensitive than is recommended by current standards^8,20,24^. Furthermore, considering that non-contrast CT scan cannot be coupled to the decellularization system itself, it requires that the organ be removed from the decellularization process in order to analyze it.

Hulsman *et al*.^41^, in turn, showed that online quality control of rat heart decellularization, including the association between the variables organ biomass, DNA, and protein ratios from the perfusate assessment, can prevent excessive exposure to decellularization reagents. Considering biomass, similarly to our findings, they found significant differences between Native dry weight and Decellularized dry weight; their remaining ECM represented around 16% of native dry biomass, while we found an average of 13% (±3 SD; p < 0.001). In contrast to the cited study, we also analyzed inefficient decellularizations, and found no significant differences in ECM biomass proportions between the Complete Decellularization and Incomplete Decellularization groups (Supplementary Fig. 1c). In the same work by Hulsman *et al*., the total DNA content of all the perfusates demonstrated only around 47% of DNA discharge, suggesting that the future development of a mathematic model to description of DNA discharge behavior may be preferable for determining the extent of genetic material removal, rather than direct calculation from the perfusate. It is observed, therefore, that the identification of cell removal requires multivariate analysis, incorporating not only the DNA discharge, but also the optical profile, which can be achieved when combined with our optical monitoring system.

This approach can be easily coupled with the presented platform, improving the algorithm by generating a solid database and allowing for valuable adjustments, since it presents a good opportunity to evaluate the methodological process. Our system also provides the necessary sensitivity, offering a mathematical model based on the optical profile that enables discrimination between samples with DNA concentration variations of 50 ng.mg^−1^of dry weights. Our system allows inefficient cell removal to be identified in the first hours; it also enables simultaneous nondestructive protocol quality control within the decellularization system itself, for which only simple electronic components are required to couple the system with other modules and adapt it to other protocols. This approach can therefore extend the comprehensiveness of the decellularization process, as it can be made available to any research laboratory, and can be used alongside other alternative techniques, contributing to the development of tissue engineering.

At present, the system is being tested with different organs, with promising results in mouse livers (Supplementary Fig. 5) and rat kidneys. A limitation of our system is that it needs to be adapted for swine or human organs, but it is believed that its physical principles can be applied to larger organs.

## Conclusion

The proposed and estimated four-parameter logistic nonlinear regression model had explanatory power in the modeling decellularization process when evaluated by optical profile, and demonstrated potential for use as a gold standard method for evaluating cellular removal with statistically significant evidence, even without any removal of background noise, which is needed to improve the results. Given that standardization and visual observation of white transparency cannot guarantee complete decellularization, the non-invasive technique presented here is useful for indicating incomplete decellularization at the beginning of the process, according to the DNA criteria. This, in turn, can help prevent further experimental complications due to inefficient decellularization. The sensor can also assist in methodological study, since it is evidenced that each heart has an individual and specific decellularization optical profile. The platform also demonstrated great potential to improve the information extracted, and to provide in-depth analysis of the process when used with different photo-detectors for specific wavelengths, or to explore molecules with auto-fluorescence properties. This improvement will produce useful data for the development of algorithms, and could lead to a new standard for organ decellularization, promoting an exponential advance in tissue bioengineering research.

## Methods

### Sensor development

The optical sensor applied to the heart decellularization consists of a two-module system coupled to an adjustable base at three axes (x, y and z) for proper organ alignment (Fig. 2a). The first module has a wide-spectrum 12 W high brightness light emitting diode source (LED). On the opposite side is the second module; a data acquisition system consisting of a photodiode (Hamamatsu S6036, 320–1100 nm spectral range) and a single-plate free hardware electronic prototyping platform connected to the computer, in which the programming software Matlab® records the data and draws the graph simultaneously. The organ is fixed at the center of the structure, where it is perfused with decellularization reagents. The needle is connected to a hose through which the organ is perfused with the decellularization reagents. At one side of the mobile base are the base adjustment knobs, which can regulate the height, proximity of the light source to the organ, and alignment of the “detector - organ - light” components. After alignment, the program is started, and records the voltage values detected by the photodiode. The liquid perfusing the organ is channeled into a receptacle connected to a discard container by a hose. The whole system is housed inside a dark chamber to isolate it from ambient light (patent application INPI BR 10 20170091872). The LED light source was used to avoid heating the surrounding environment and prevent the emission of ultraviolet radiation. The LED spectrum is shown in Fig. 3(e).

For the spectral analysis of reflectance and transmittance, two silica optical fibers were attached to the mobile base, positioned at 180° and 90° to the optical source. The decellularization process was monitored with a fiber optic spectrophotometer (Ocean Optics USB2000).

### Data acquisition

Decellularizations were performed inside the structure, with the heart supported between the light source and the photo detector during perfusion of reactants. The software recorded the voltage produced by the photodiode from the LED through the organ, during the surfactant sodium dodecyl sulfate (SDS) perfusion. The sensor was standardized by maintaining a distance of 5 cm between lamp and the organ, by positioning the organ with the sternocostal side towards the photo detector and the diaphragmatic side towards the light source, and centralization of the organ, with the lowest possible voltage value. A value of 0 V indicates the absence of photons detected by the sensor, and 5 V corresponds to the saturation voltage of the system. Intrinsic variations in the light source were monitored for 20 hours (Supplementary Fig. 4a). The voltage values of water perfusion through a previously decellularized organ were collected over a period of 1 hour (Supplementary Fig. 4b). Prior to each data recording, the light source was switched on for about 2 hours until stabilization (Supplementary Fig. 4c).

Data were captured from the reflectance and transmittance spectral analysis with the fiber optic spectrophotometer; this was done every 30 minutes for the first two hours and then every 1 hour until the end of the decellularization. The experiment was performed in triplicate, with three values per measurement.

### Animals

The experiments were approved by the Ethics Committee on the Use of Animals of the Federal University of Espírito Santo (CEUA-UFES) under license No. #033–2014, and were carried out in accordance with the National Council for Animal Control and Experimentation (CONCEA-Brazil). A total of 20 hearts were extracted from young male Wistar rats, with an average weight of 295 ± 29 g, aged between 08 and 10 weeks. The animals were reared in the animal facility at the Center of Health Sciences of the Federal University of Espírito Santo (UFES). The animals received water and food *ad libitum* and were maintained under a controlled light/dark cycle of 12 hours, temperature of 22 ± 2 °C, and relative humidity of 60 ± 5% until euthanasia.

### Organ decellularization

The animals were heparinized (2UN.g^−1^ Hepamax-s, Blausiegel®) and anesthetized with Ketamine (90 mg/kg Ketamine-Vetnil®) and Xilazine (10 mg.kg^−1^ Rompun-Bayer®) intraperitoneally. With the animal in the dorsal decubitus position, an incision was made under the sternum bone, and the hearts were extracted and frozen at −80 °C for up to 48 hours. The organs were then progressively thawed for 12 hours at −20 °C and 12 hours at 4 °C. Next, adjacent tissues were removed and the organ was weighed. The average organ mass was 1.2 ± 0.11(SD) grams. The hearts were fixed to a support and the ascending aortas were accessed for retrograde perfusion of sodium dodecylsulfate solution (SDS), by aortic cannulation and connection to a peristaltic pump (Gilson®), which promoted antegrade perfusion through the coronary arteries. All the hearts were decellularized according to the protocol previously published by Guyette *et al*.^11^. In summary, sodium phosphate buffer (PBS, pH 7.3) was perfused for 30 minutes at an initial flow rate of 2 mL.min^−1^ and a coronary perfusion of SDS(1% w.v^−1^) for about 12 hours with controlled pressure at 80 mmHg (±10). All organs reached white transparency after SDS perfusion and did not present any areas of tissue remaining in the scaffold. Then, distilled water was perfused for 30 minutes, Triton-X-100 reagent (1% v.v^−1^) for 30 minutes, and, finally, PBS was used for 1 hour. At the end of the process, the maximum flow rate was 4 mL.min^−1^. After the procedure, the organs were placed in a freezer at −80 °C freezer, lyophilized (L101 - Liotop®), and then stored at −80 °C until DNA quantification. Native hearts were also lyophilized and stored at −80 °C. Each individual decellularization was categorized as Complete Decellularization or Incomplete Decellularization only after the DNA quantification result.

### DNA quantification

Total DNA content was isolated by a standard salt extraction protocol^42,43^. Briefly, the lyophilized organs were cut; the fragments were weighed (up to 14 mg), finely perforated and packed in sterile micro tubes. Subsequently, the steps of lysis, precipitation and purification were performed. In the lysis step, 410 µL of extraction buffer (1% TRIS-1M, 2% NaCl-5M, 2% EDTA-0.5 M and ultrapure water), 80 µL of SDS 10% and 15 μL of proteinase K solution (20 μg.μL^−1^) were added, incubating the solution overnight at 55 °C. In the precipitation step, the micro tubes were centrifuged and the supernatant was transferred to a new tube. Then, 180 μL of NaCl (5 M) were added, homogenizing and centrifuging the tube again. Subsequently, the supernatant was transferred to a new tube, to which was added 800 µL of isopropanol, previously stored at −20 °C. After homogenization and further centrifugation, the supernatant was discarded and 250 µL of 80% ethanol was added, repeating the operation for purification. The tube was then centrifuged, the supernatant discarded, and the remaining ethanol removed by evaporation at 55 °C. The pellet was diluted in 25 µL and 50 µL of ultrapure water for decellularized and control samples, respectively. Finally, the micro tubes were stored at between 4 and 8 °C overnight for further spectrophotometric reading (Nanodrop® 2000-Therm Scientific) and DNA quantification per mg of lyophilized tissue. After decellularization and DNA quantification, each individual organ was categorized as Complete Decellularization or Incomplete Decellularization, according to the DNA concentration result. The efficacy of decellularization was based on minimal criteria of residual DNA (<50 ng DNA per mg of dry matrix^8^ and 96% DNA removal^11^. The Complete Decellularization group included decellularizations with a maximum residual DNA of 4.6%, representing a DNA concentration of 27(±19 SD) ng.mg^−1^ of dry weight. The Incomplete Decellularization group comprised decellularizations with less than 95.4% of DNA removal, represented by a concentration average of 301 (±140 SD) ng.mg^−1^ of dry weight.

### Collagen quantification

Collagen content was determined according to the standard protocol described previously^44,45^. Briefly, fragments of lyophilized hearts from the Native (n = 4), Complete Decellularization (n = 4) and Incomplete Decellularization (n = 4) groups were transferred to15 mL conical plastic tubes for alkaline hydrolysis with 50 μL of sodium hydroxide solution (7 M). One sample without tissue was used as blank. Then, 50 μL of sulfuric acid (3.5 M) was added to the hydrolyzate and diluted to 1 mL with distilled water. From this solution, 50 μL was transferred to a microtube and 450 μL of Chloramine T (0.0025 M) was added. After 25 minutes at room temperature, 500 μL of Erlich reagent (0.5 M) was added and the solution was heated in water bath for 15 minutes (60 °C). Finally, the absorbance at 560 nm was measured using a microplate reader (Varioskan Flash Model, Thermo Fisher). Hydroxyproline standards were used to construct the standard curve (Sigma Aldrich, USA) with concentrations ranging from 2.5 to 1,300.0 μg.mL^−1^. The absorbance values were calculated by the equation Y = 0.003474 * X + 0.06491.

### Histology

Essentially, the histology tissue preparation included the following steps: fixation, dehydration, inclusion, microtomy, assembly and staining. Thus, three samples from each group were fixed in paraformaldehyde solution (4%) with sodium phosphate buffer (PBS, pH 7.4) for 48 hours. The samples were then dehydrated with sequential 30-minutes baths in 70%, 95% and 100% ethanol solutions. For the inclusion step, histological infiltration with Historesin Embedding Kit (Jung, Germany) was performed following the manufacturer’s protocol. At microtomy, 5 μm sections were cut and stained with Haematoxylin and Eosin (H&E).

### Statistics

DNA and collagen content differences among studied groups were compared by Welch one-way analysis of variance (ANOVA) for heteroscedastic data and p-values < 0.05 were considered statistically significant. All pairwise comparisons were performed by the Bonferroni *post-hoc*, which was also applied to the heteroscedastic data. The values are expressed as means ± SD (standard deviation). For the voltage measured over time, a mixed non linear model was fitted using the software R (nlme version 3.1 from Pinheiro and Bates, 2000)^28^. The model includes a random effect for each organ, to take into account that the measures evaluated in the same organ are correlated. A four-parameter logistic nonlinear regression model^27,29^ obtained the best fit, and is defined as follows:

$$y=\frac{A1-A2}{1+{(\frac{x}{{x}_{0}})}^{s}}+A2$$

where y is the expected voltage (volts) at time x (seconds), A1 represents the initial voltage, A2 represents the maximum asymptote or the final voltage, *s* corresponds to the curve slope and x_0_ to the inflection point or the halfway time between the minimum and maximum voltage asymptotes. First, this model was evaluated in a descriptive analysis of time series plots performed using the Origin pro8 software, where the proposed non linear model was chosen because all its parameters are interpretable and also because it fitted well to the observed data, with the same S-curve identified in the individual profile of the voltages over time. Other models based on sigmoidal functions with 3 parameters (y = a/(1 + exp(−k*(x − xc))) and 4 parameters (y = A1 + (A2 − A1)/(1 + 10^(LOGx0-x)^*p)) presented worse fits. The adjusted R-squared of our model was 0.975.

One-way ANOVA followed by the Bonferroni method was used to the comparison of mean collagen content among groups, (n = 4 per group) and p-values < 0.05 were considered statistically significant. The values are expressed as means ± SD (standard deviation).

## Supplementary information

Supplementary Figure 1,2,3,4 and 5
