# Supplementary material for: A non-linear mathematical model using optical sensor to predict heart decellularization efficacy
Source: Sci Rep. 2019 Aug 21;9:12211. doi: 10.1038/s41598-019-48659-3 (PMC6704168; doi:10.1038/s41598-019-48659-3)
Supplement: Supplementary file 1 — Supplementary Figure 1,2,3,4 and 5 [file 41598_2019_48659_MOESM1_ESM.pdf]

A non-linear mathematical model using optical sensor to predict heart decellularization efficacy

Rayssa Helena Arruda Pereira, Adilson Ribeiro Prado, Luiz Felipe Castello Del Caro, Tadeu Ériton Caliman Zanardo, Airlane Pereira Alencar, Breno Valentim Nogueira

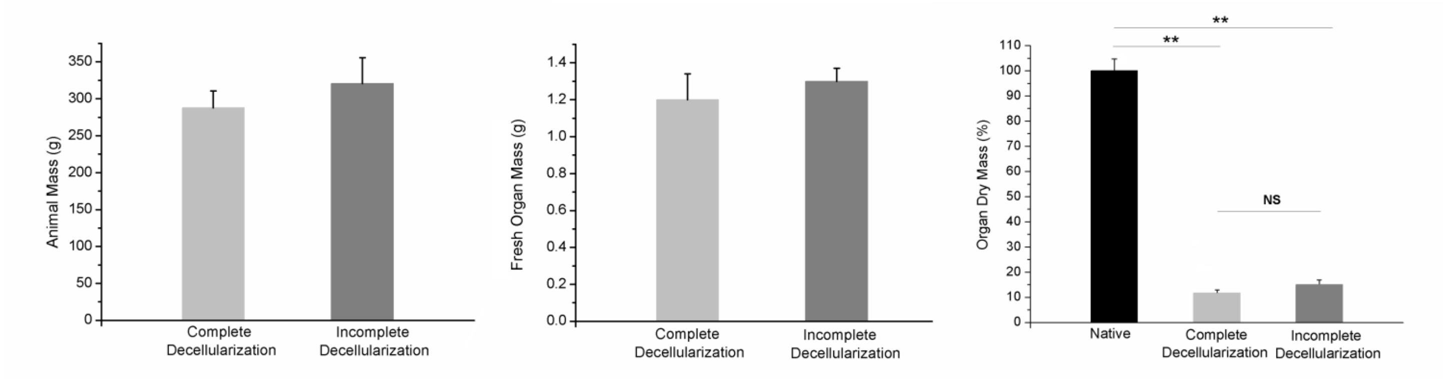

Supplementary Figure 1. a and **b,** Distribution of animal masses and fresh organs masses between groups (Complete Decellularization, n=6; Incomplete Decellularization, n=8; unpaired two-tailed t-test.). **c,** Representation of hearts dry weight from the Native, Complete Decellularization and Incomplete Decellularization groups (n=4 per group; Welch one-way ANOVA) $**p<0.05$ *vs.* Native.

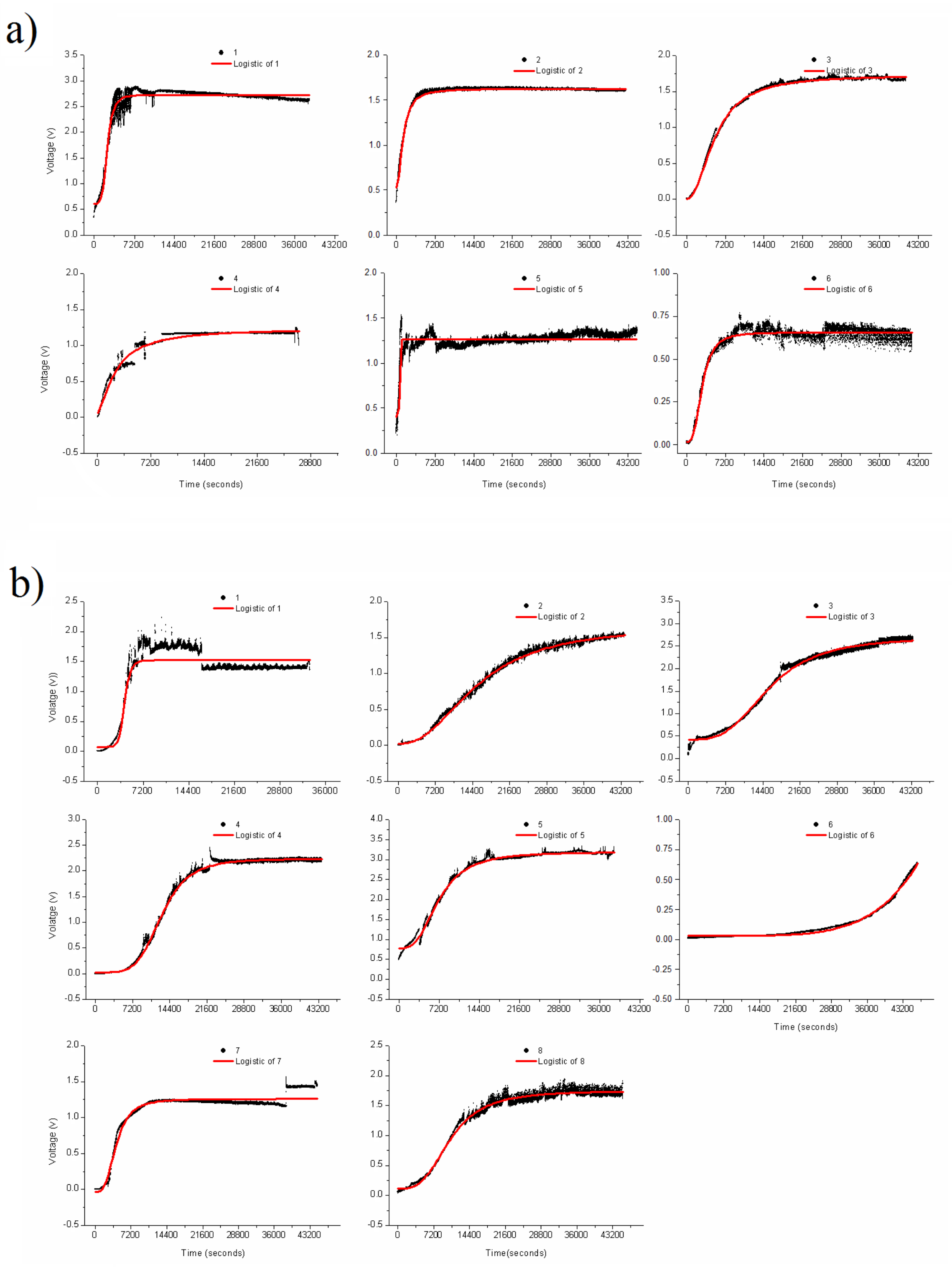

Supplementary Figure 2.a, Original “Voltage x Time” curves in black with their respective fits shown in red for the Decellularized Group. **b,** Original “Voltage x Time” curves shown in black with their respective fits in red for the Incomplete Decellularization group. The curves were fitted by a four-parametric non linear model described by Gadagkar and Call [27].

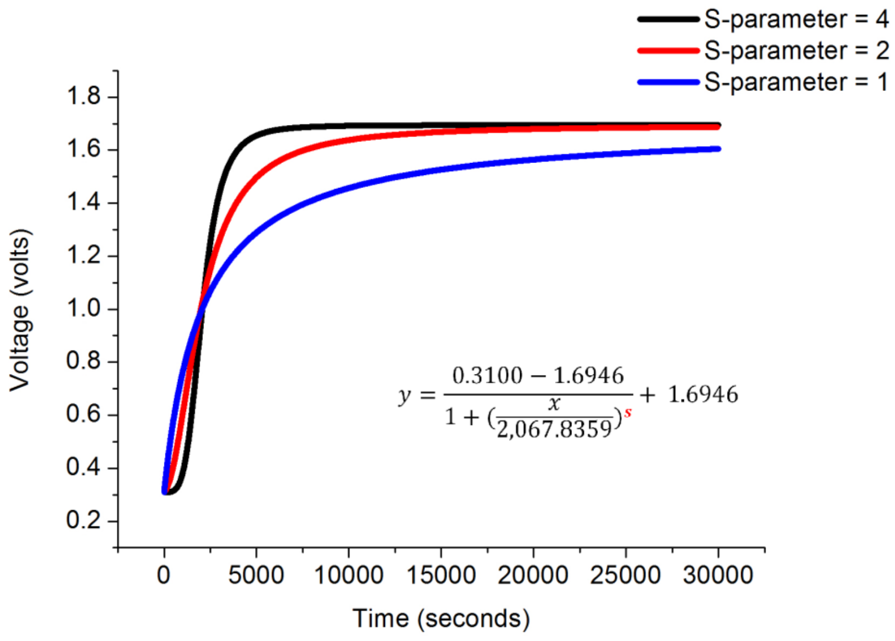

Supplementary Figure 3. Representation of the influence of the *s* parameter on the shape of the curve. Here, the values of the three remaining parameters (A1, A2 and x_0) of the Decellularized group were set, while the *s* parameter or values 1, 2 and 4 varied.

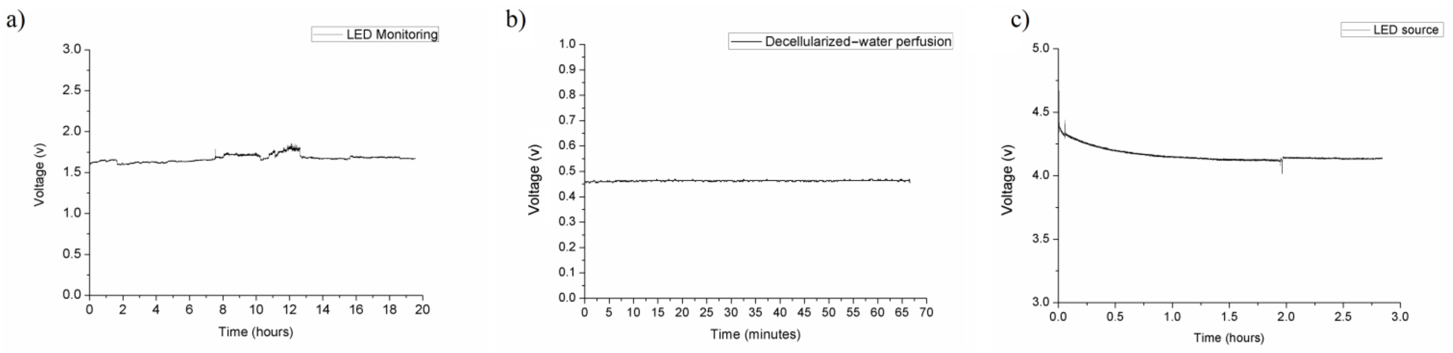

Supplementary Figure 4. Characterization of the system signal. **a,** The intrinsic variations of the light source monitored for 20 hours. **b,** Voltage measured during water perfusion through a previously decellularized heart, monitored for approximately 1 hour. **c**, Light source behavior from the start time until stabilization.

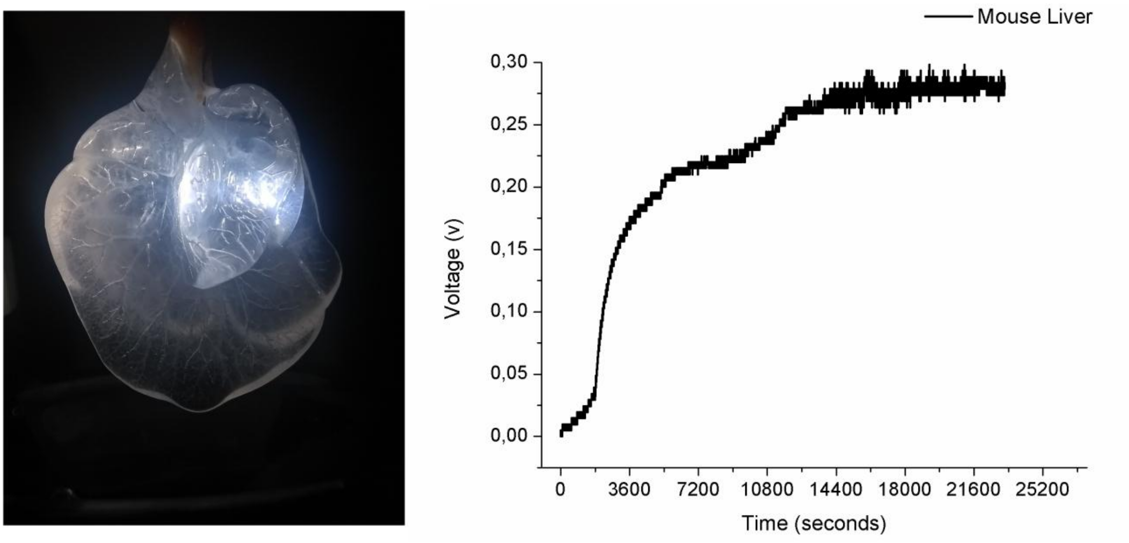

Supplementary Figure 5. Liver decellularization and voltage-time graphic record. Image of mouse decellularized liver after seven hours of SDS (1% w.v^{-1}) perfusion, and typical voltage-time graphic record.
